# Evaluation of the reentry vulnerability index to predict ventricular tachycardia circuits using high-density contact mapping

**DOI:** 10.1016/j.hrthm.2019.11.013

**Published:** 2020-04

**Authors:** Michele Orini, Adam J. Graham, Neil T. Srinivasan, Fernando O. Campos, Ben M. Hanson, Anthony Chow, Ross J. Hunter, Richard J. Schilling, Malcolm Finlay, Mark J. Earley, Simon Sporton, Mehul Dhinoja, Martin Lowe, Bradley Porter, Nicholas Child, Christopher A. Rinaldi, Jaswinder Gill, Martin Bishop, Peter Taggart, Pier D. Lambiase

**Affiliations:** ∗ Institute of Cardiovascular Science, University College London, London, United Kingdom; † The William Harvey Research Institute, Queen Mary University of London, London, United Kingdom; ‡ Electrophysiology Department, Barts Heart Centre, St Bartholomew's Hospital, London, United Kingdom; § School of Biomedical Engineering and Imaging Sciences, King's College London, London, United Kingdom; ¶ Department of Mechanical Engineering, University College London, London, United Kingdom; ‖ Department of Cardiology, Guys and St Thomas' NHS Trust, London, United Kingdom

**Keywords:** Ablation, Activation time, Reentry vulnerability index, Repolarization time, Substrate mapping, Ventricular tachycardia

## Abstract

**Background:**

Identifying arrhythmogenic sites to improve ventricular tachycardia (VT) ablation outcomes remains unresolved. The reentry vulnerability index (RVI) combines activation and repolarization timings to identify sites critical for reentrant arrhythmia initiation without inducing VT.

**Objective:**

The purpose of this study was to provide the first assessment of RVI’s capability to identify VT sites of origin using high-density contact mapping and comparison with other activation-repolarization markers of functional substrate.

**Methods:**

Eighteen VT ablation patients (16 male; 72% ischemic) were studied. Unipolar electrograms were recorded during ventricular pacing and analyzed offline. Activation time (AT), activation–recovery interval (ARI), and repolarization time (RT) were measured. Vulnerability to reentry was mapped based on RVI and spatial distribution of AT, ARI, and RT. The distance from sites identified as vulnerable to reentry to the VT site of origin was measured, with distances <10 mm and >20 mm indicating accurate and inaccurate localization, respectively.

**Results:**

The origins of 18 VTs (6 entrainment, 12 pace-mapping) were identified. RVI maps included 1012 (408–2098) (median, 1st–3rd quartiles) points per patient. RVI accurately localized 72.2% VT sites of origin, with median distance of 5.1 (3.2–10.1) mm. Inaccurate localization was significantly less frequent for RVI than AT (5.6% vs 33.3%; odds ratio 0.12; *P* = .035). Compared to RVI, distance to VT sites of origin was significantly larger for sites showing prolonged RT and ARI and were nonsignificantly larger for sites showing highest AT and ARI gradients.

**Conclusion:**

RVI identifies vulnerable regions closest to VT sites of origin. Activation-repolarization metrics may improve VT substrate delineation and inform novel ablation strategies.

## Introduction

Recurrence rates of ventricular tachycardia (VT) in structural heart disease remain suboptimal, with 50% on average for a first-time catheter ablation highlighting the need for more effective ablation strategies.^1^ Although VT induction and activation/entrainment mapping is the preferred method for identifying the circuit, it often cannot be performed due to instability of the VT itself or hemodynamic compromise.^2^ Substrate ablation strategies have been proposed and are based on electrogram features related to signal morphology or local conduction parameters.^3^^,^^4^

Even though the earliest research in the field identified spatiotemporal repolarization dynamics as one of the fundamental factors modulating vulnerability to reentry,^5^ current clinical practice relies almost exclusively on conduction-related parameters to identify target sites. A prerequisite for reentry is unidirectional block whereby an activation wavefront blocks at a region of late repolarization where tissue is still refractory circumvents the area of block through slow conducting pathways and reenters the proximal region. The ability to reenter the proximal region depends not only on the conduction delay around the blocked area but also on the timing of the returning wavefront relative to completion of repolarization and hence reexcitability in the proximal region.^6^ This is the basis of the reentry vulnerability index (RVI),^6^^,^^7^ an activation-repolarization metric that provides a point-by-point quantification of the likelihood of reentry and enables functional VT substrate delineation.

Previous mechanistic studies^6^, ^7^, ^8^, ^9^ based on *ex vivo* animal data and computational models have confirmed the link between RVI and sites of VT initiation. Preliminary observations on retrospective data utilizing noncontact mapping technology in selective right ventricular (RV) disorders have shown encouraging results.^10^ However, RVI’s potential as a clinical tool to localize critical sites for VT initiation has never been formally assessed using state-of-the-art high-density mapping. The aim of this study was to relate the vulnerable region delineated by RVI to the VT site of origin (VT-SoO) and compare RVI to other spatiotemporal metrics of activation and repolarization.

## Methods

### Concept and quantification of RVI

**Figure 1:**
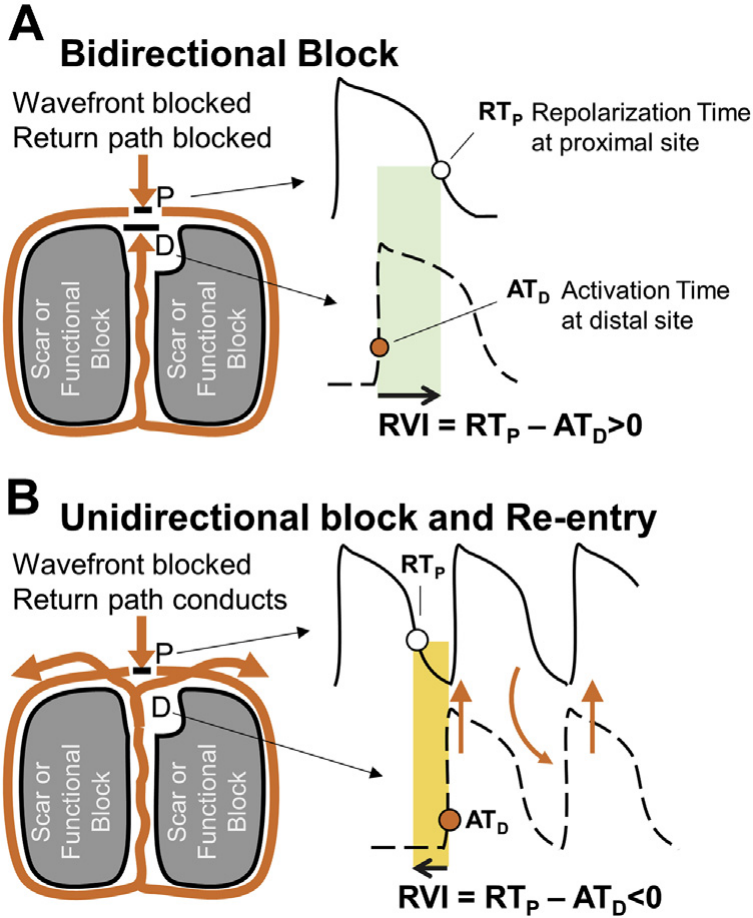
Theoretical model underpinning the reentry vulnerability index (RVI). **A:** A re-entrant wavefront is blocked (RT at point P longer than AT at point D = large RVI) **B:** A re-rentrant wavefront conducts and enables a re-entry (RT at point P shorted than AT at point D = negative RVI). See “Methods” for a detailed description of this figure. Similar diagrams can be found in Coronel et al,^6^ Child et al,^7^ and Martin et al.^10^

The RVI concept is illustrated in Figure 1. Figure 1A shows the case of bidirectional block and no reentry. An activation wavefront (orange line) arrives at a region that is refractory and blocks (point P). The wavefront travels around the area of block (orange line) and arrives back at the distal side of the block (point D), which still is refractory and blocks in the reverse direction (bidirectional block). Bidirectional block occurs because repolarization time (RT) at point P is longer than activation time (AT) at point D. Figure 1B illustrates the case where reentry occurs. The returning wavefront arrives back at the initial site of block (point D) when the region proximal to it has regained excitability. The returning wavefront now is able to propagate back to the proximal region and complete a reentrant circuit. Reentry occurs because RT at point P is shorter than AT at point D. RVI is represented by the interval between AT at the distal site D and repolarization at the proximal site P, that is, RT_P_-AT_D_, shown as shaded areas in Figure 1. A shorter RVI (Figure 1B) is more likely to be associated with reentry than a longer RVI (Figure 1A).

### Patients and procedures

Patients with structural heart disease undergoing catheter ablation for VT were prospectively recruited at the Barts Heart Centre and St Thomas’s Hospital (London, United Kingdom). Patients gave informed consent for inclusion into VT mapping research approved by the local Research Ethics Committee. Cardiac mapping was performed with either CARTO (PentaRay and DecaNav, Biosense Webster Inc., Diamond Bar, CA) or EnSite Precision (HD Grid, Abbott, Des Plaines, IL) (Table 1). The mapping data were included in the study if VT-SoO was identified and a sufficiently dense substrate map was produced.

**Table 1:** Patient information

	Sex	Age (y)	Etiology	VT-SoO	EAM	Catheter	Pacing interval (ms)	Pacing type	Points on map (n)	Dist to VT-SoO (mm)
1	M	69	IHD	PM	CARTO	PentaRay	500	S_1_S_1_	4190	8.3
2	F	34	ARVC	ENT	CARTO	PentaRay	460	S_1_S_1_	2256	17.9
3	M	71	IHD	PM	CARTO	PentaRay	360	S_1_S_2_	2560	4.9
4	F	52	ARVC	PM	CARTO	PentaRay	360	S_1_S_1_	1312	5.2
5	M	79	IHD	ENT	CARTO	PentaRay	380	S_1_S_2_	260	4.8
6	M	55	IHD	PM	CARTO	PentaRay	360	S_1_S_2_	1625	8.2
7	M	70	IHD	PM	CARTO	PentaRay	360	S_1_S_2_	370	16.5
8	M	73	ARVC	PM	CARTO	PentaRay	360	S_1_S_2_	328	5.2
9	M	22	ARVC	PM	CARTO	DecaNav	1000	S_1_S_1_^BV^	4325	0.0
10	M	65	IHD	ENT	Precision	HD Grid	360	SE	1341	33.9
11	M	68	IHD	PM	Precision	HD Grid	360	SE	1304	13.8
12	M	50	IHD	PM	Precision	HD Grid	360	SE	4328	10.7
13	M	61	IHD	ENT	Precision	HD Grid	325	SE	719	1.9
14	M	65	IHD	PM	Precision	HD Grid	360	S_1_S_2_	356	3.4
15	M	77	IHD	PM	Precision	HD Grid	400	SE	389	5.0
16	M	60	IHD	PM	Precision	HD Grid	390	SE	466	1.7
17	M	52	IHD	ENT	Precision	HD Grid	360	SE	511	3.1
18	M	65	IHD	ENT	Precision	HD Grid	400	SE	667	2.5
	89% M	65 (53-70)	72% IHD	67% PM	50% CARTO	50% HD Grid	360 (360–398)	44% SE	1012 (408–2098)	5.1 (3.2–10.1)

Pacing maneuvers to determine the ventricular tachycardia site of origin (VT-SoO) were either entrainment (ENT) or pace-mapping (PM). Electroanatomic mapping (EAM) systems were CARTO or EnSite Precision. Pacing types were S_1_S_1_, S_1_S_2_, or sensed extras (SE).

ARVC = arrhythmogenic right ventricular cardiomyopathy; Dist to VT-SoO = distance between VT-SoO and the nearest site showing the lowest reentry vulnerability index; IHD = ischemic heart disease; Points on map = number of unipolar electrograms per map; S_1_S_1_^BV^ = biventricular pacing.

Mapping was performed during ventricular pacing from the RV apex. If pacing was well tolerated, a train of 5 S_1_ paced beats was delivered, followed by an S_2_ beat at a short coupling interval (Table 1) to induce slow conduction necessary for RVI calculation.^7^ If continuous pacing was poorly tolerated, either a train of 3 short coupled beats was delivered or a single paced beat was delivered shortly after sensing the R wave of a sinus beat. In 1 patient with severely impaired function, biventricular pacing was delivered at a normal rate (60 bpm). The pacing procedure was continuously repeated and data collected on the postextrasystolic beat using standard criteria to enable high-density sequential mapping.

In case of hemodynamically tolerated VTs, identification of VT-SoO was defined by either entrainment or termination of VT during ablation. For unstable VTs, VT-SoO was identified using pace-mapping with an average correlation coefficient between the 12-lead electrocardiogram of VT and the paced beat ≥90%.^11^ If multiple sites for the same VT were identified with pace-mapping, the site providing the highest correlation was retained.

Ablation was delivered at the VT-SoO and at other sites to achieve substrate modification using standard criteria^2^ and was not based on RVI, which was computed offline.

### Data analysis

**Figure 2:**
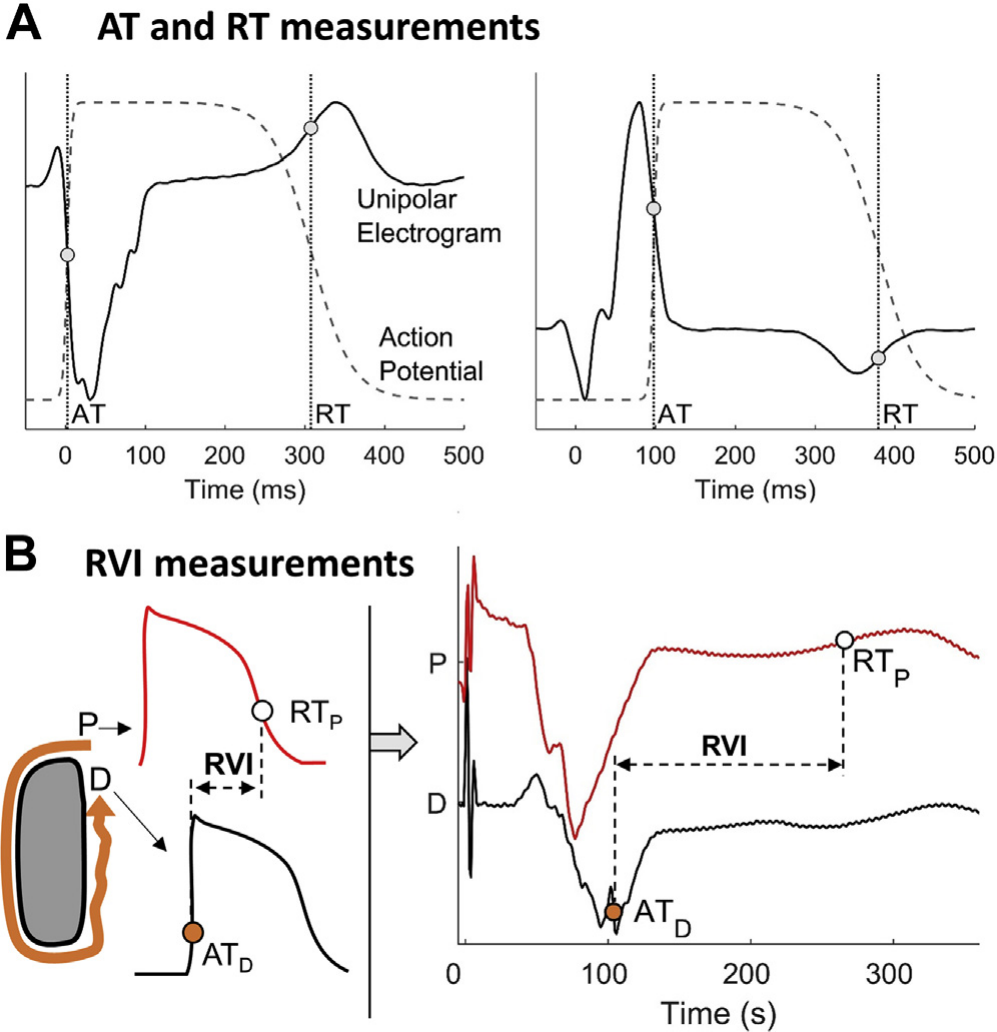
Computation of spatial activation-repolarization metrics. **A:** Stylized action potentials and unipolar electrograms showing standard measurements of activation (AT) and repolarization (RT) times. **B:** Conceptual model for reentry vulnerability index (RVI) measurement **(left)** and RVI measurements using recorded unipolar electrograms **(right)**. D = distal site; P = proximal site.

Unipolar electrograms were recorded with bandpass filters set at 0.05–500 Hz and were exported along with anatomic data for bespoke offline analysis in MATLAB (The MathWorks, Natick, MA), which included stringent criteria for selecting only beats showing very similar activation/repolarization patterns (Supplementary Methods). AT, RT, and activation–recovery interval (ARI) were measured following standard definitions^12^^,^^13^ (Figure 2) using automatic robust algorithms developed and tested during the course of previous studies.^10^^,^^14^, ^15^, ^16^, ^17^ Markers were revised using bespoke graphical user interfaces and semiautomatic correction, which involved performing automatic annotation within manually adjusted windows of interest. Correction was limited to isolated outliers to reduce arbitrary annotation and ensure reproducibility.

### Localization of sites vulnerable to reentry

**Figure 3:**
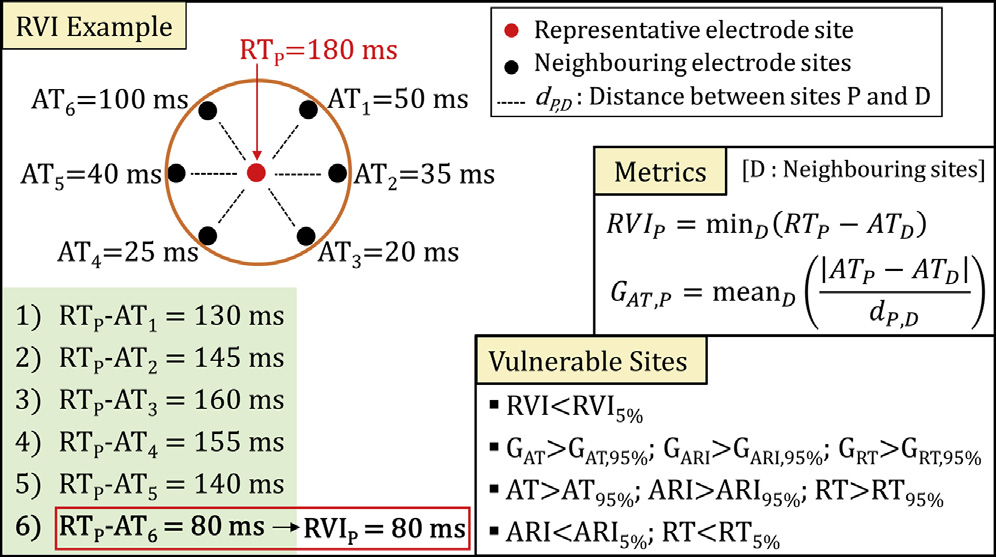
Computation of reentry vulnerability index (RVI) and spatial activation-repolarization metrics. **Left:***Red dot* represents a cardiac site P for which RVI is measured. *Black dots* represent neighboring cardiac sites within a searching radius R. As shown in the example in the box, RVI is the shortest interval between AT at neighboring sites and RT at site P (RT_P_-AT_D_). **Right:** Formula for RVI and local gradients measurement. Local gradients of ARI and RT are measured in the same way. **Bottom right:** Criteria for identifying vulnerable sites to reentry. Abbreviations as in Figure 1, Figure 2.

The algorithm for RVI mapping operates as follows. For each cardiac site, neighboring electrode sites are identified within a searching radius R = 8 mm. The intervals between RT at a given site P and AT at neighboring sites D (ie, RT_P_-AT_D_) are measured. The shortest of these intervals represents RVI at site P. RVI was thereby obtained for each electrode site in the mapped area. This process is summarized in Figure 3, with an example provided.

For comparison, local gradients of AT, ARI, and RT, which provide a quantification of local activation and repolarization heterogeneity, also were computed (Figure 3).

The distance between VT-SoO and the nearest of the following sites was measured in order to assess the capability of localizing critical sites for VT initiation:

• Sites showing the lowest RVI, that is, RVI <5th percentile of RVI values. RVI values ≥300 ms were excluded from those considered as vulnerable to reentry even if within the lowest 5%
• Sites showing the largest AT, RT, and ARI gradients, that is, sites for which local gradients were >95th percentile of their distributions
• Sites showing the longest AT, that is, AT >95th percentile of AT values
• Sites showing the longest RT and ARI, that is, sites for which RT and ARI were > 95th percentile of their distributions
• Sites showing the shortest RT and ARI, that is, sites for which RT and ARI were <5th percentile of their distributions

### Statistical analysis

Data are reported as median (1st—3rd quartiles). The Wilcoxon signed-rank test was used for comparing distances between VT-SoO and vulnerable sites identified by different markers, with *P* <.05 indicating significance.

## Results

In total, 18 patients were included in the study. Baseline characteristics are listed in Table 1. Patient age was 65 (52–70) years, and 16 were male. Pathologies included ischemic heart disease (n = 14 [78%]) and ARVC (arrhythmogenic right ventricular cardiomyopathy) (n = 4 [22%]). The cycle length of the beat preceding the mapped beat was 360 (360–398) ms, and electroanatomic maps for RVI calculation included 1012 (408–2098) unipolar electrograms (Table 1). Some patients presented >1 VT morphologies at the time of the procedure, but in all cases the site of origin of only 1 VT was identified and confirmed by either entrainment (n = 6) or pace-mapping (n = 12) (Table 1). Other VTs were not mapped either because they were thought not to be clinical or because a substrate ablation approach was preferred. This was often the case, as VT was unstable in two-thirds of patients.

Acute VT inducibility was tested at the end of the procedure in 12 patients (67%) but was considered inappropriate in the other 6 patients (33%) because of hemodynamic compromise. None of the mapped VTs was inducible at the end of the procedure, whereas in 2 (16%) patients a different VT morphology was induced.

During median follow-up of 16.2 (7.1–20.3) (minimum–maximum) months, 50% of patients (75% of ARVC, 43% of ischemic patients) suffered a recurrence, defined as any therapy from the implantable device, including antitachycardia pacing, heart transplant, or death from any cause.

### Assessment of RVI in relation to VT-SoO

**Figure 4:**
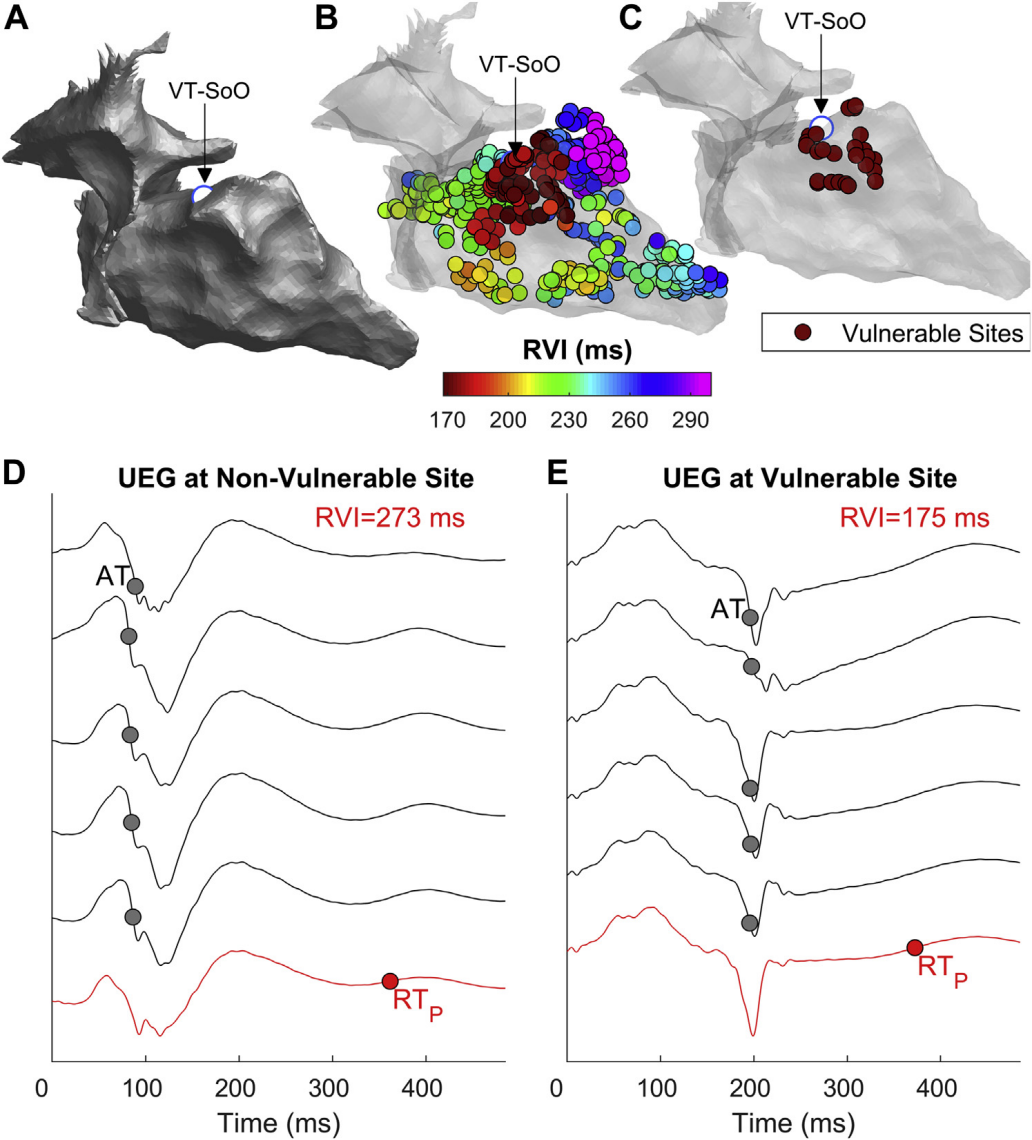
Example of reentry vulnerability index (RVI) identifying vulnerable sites close to an entrained ventricular tachycardia (VT). **A:** Anatomic map showing the VT site of origin (VT-SoO) as a *white dot.***B:** RVI map. Each dot represents a cardiac, site and RVI is color-coded. **C:** Map showing sites with the lowest 5% of RVI values. **D, E:** Unipolar electrograms (UEGs) from an electrode site showing high **(D)** and low **(E)** RVI *(red line)* and from neighboring electrode sites *(gray). Red* and *gray circles* represent repolarization time (RT) at the site of RVI measurement (RT_P_) and activation time (AT) at neighboring sites, respectively.

**Figure 5:**
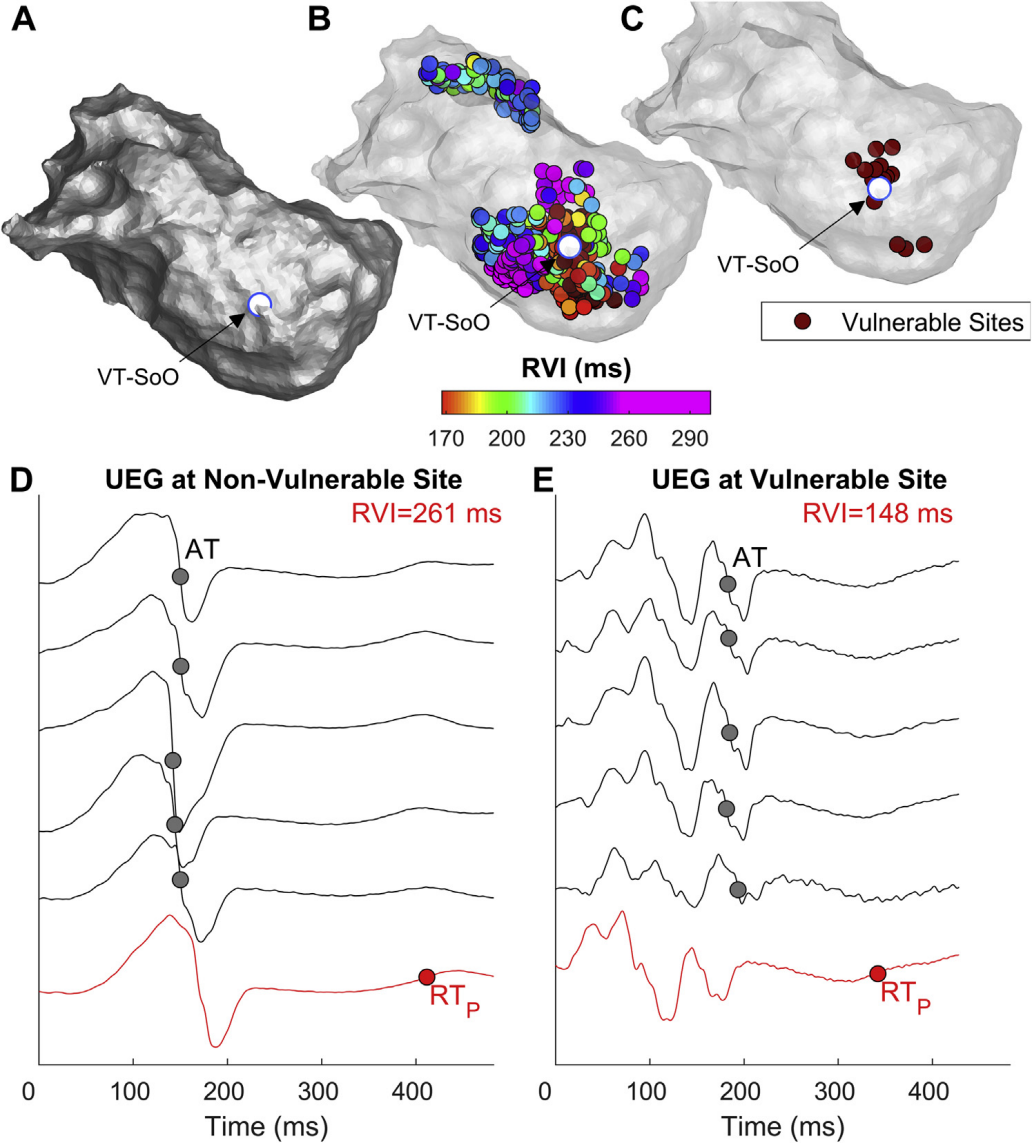
Example of reentry vulnerability index (RVI) identifying vulnerable sites close to a pace-mapped ventricular tachycardia. **A:** Anatomic map showing the VT site of origin (VT-SoO) as a *white dot*. **B:** RVI map. Each dot represents a cardiac site and RVI is color-coded. **C:** Map showing sites with the lowest 5% of RVI values. **D,E:** Unipolar electrograms (UEGs) from an electrode site showing high (D) and low (E) RVI (*red line*) and from neighboring electrode sites (*gray*). *Red* and *gray circles* represent repolarization time (RT) at the site of RVI measurement (RTP) and activation time (AT) at neighboring sites, respectively. Abbreviations as in Figure 4.

Figures 4A to 4C show RVI maps of a patient in whom VT-SoO was identified by entrainment. The sites of lowest RVI are clustered around the VT-SoO, with the closest being 3.1 mm away from it. Unipolar electrograms from electrode sites with large and low RVIs are shown in Figures 4D and 4E. Figures 5A to 5C show examples from another patient in whom the VT-SoO was identified by pace-mapping. In this case, the distance between the lowest RVI sites and VT-SoO was 3.4 mm. Some of the lowest RVI sites are distant from VT-SoO, which is expected because these may be related to a different reentrant circuit from that identified during the procedure. Unipolar electrograms are shown in Figures 5D and 5E, with low-amplitude fractionated signals recorded at a site of low RVI (Figure 5E) close to VT-SoO.

Considering all 18 VTs, the distance between the sites of lowest RVI and VT-SoO was a median of 5.1 (3.2–10.1) mm (Table 1). This was not different in ischemic and ARVC patients [4.9 (3.1–10).7 mm vs 5.2 (2.6–11.6 mm); *P* = .95] or in VTs with a site of origin identified using pace-mapping vs entrainment [5.1 (4.1–9.5 mm) vs 4.0 (2.5–17.9) mm; *P* = .82].

Similar results were obtained using searching radius R = {7, 8, 9, 10} mm, but for smaller search radii the distance between the vulnerable region and VT-SoO increased (Supplementary Figure 1).

### Comparison between RVI and other markers in relation to VT-SoO

**Figure 6:**
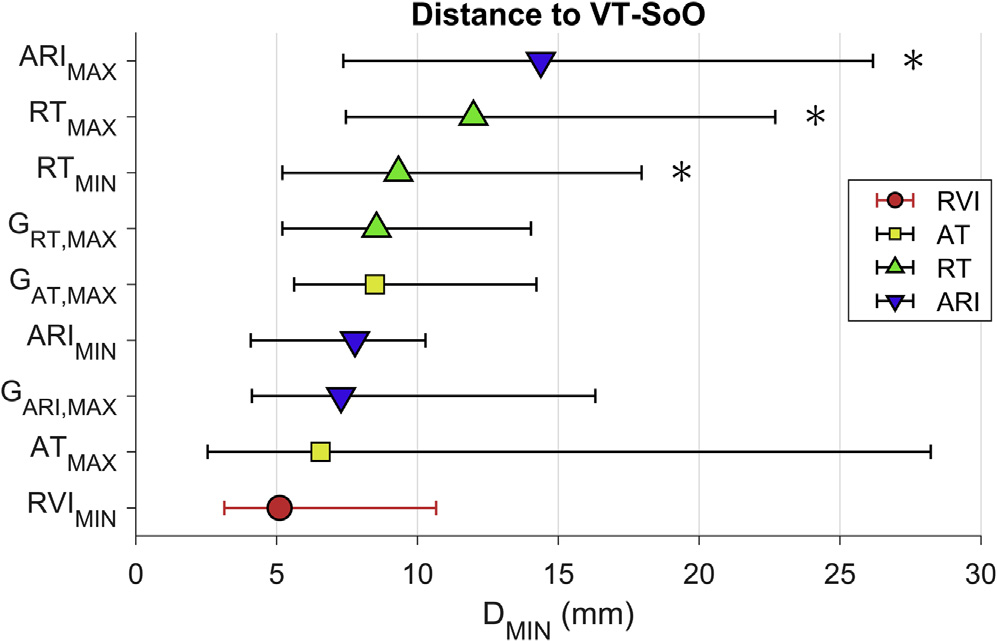
Distance between the ventricular tachycardia (VT) site of origin (VT-SoO) and the nearest vulnerable sites identified by lowest reentry vulnerability index (RVI_MIN_), largest gradients of activation time (AT) (G_AT,MAX_), largest gradients of repolarization time (RT) (G_RT,MAX_), largest gradients of activation–recovery interval (ARI) (G_ARI,MAX_), longest AT (AT_MAX_), shortest RT (RT_MIN_), longest RT (RT_MAX_), shortest ARI (ARI_MIN_), and longest ARI (ARI_MAX_). Markers indicate the median of minimum distances, and bars span the 1st–3rd quartile interval (across n = 18 VTs). *P <.05 with respect to RVI_MIN_.

The distance between VT-SoO and the lowest RVI sites was shorter than that for any other activation-repolarization marker (Figure 6). Pairwise comparisons showed that the distance to VT-SoO was significantly shorter for the lowest RVI sites than for sites showing longest RT (*P* = .020), longest ARI (*P* = .004), and shortest RT (*P* = .042). Despite showing the lowest median value as well as the lowest standard deviation across all mapped VTs, the distance to VT-SoO from the lowest RVI sites was not significantly smaller than the distance to VT-SoO from sites showing the largest local gradients of AT (G_AT_; *P* = .17), ARI (G_ARI_; *P* = .49), RT (G_RT_; *P* = .11), or from sites showing the lowest ARI (*P* = .68) and largest AT (*P* = .13) (Figure 6).

**Figure 7:**
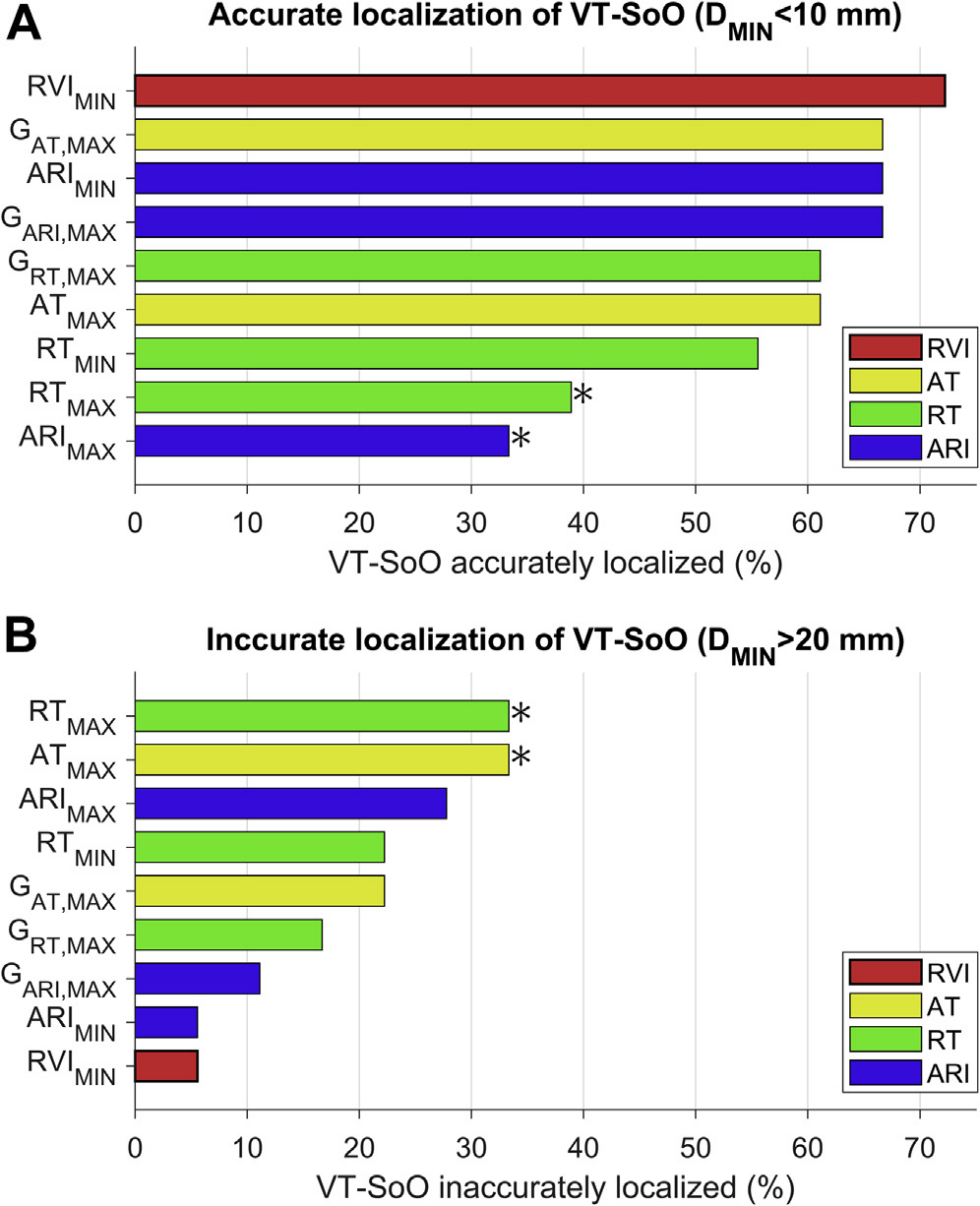
Accuracy of ventricular tachycardia (VT) site of origin (VT-SoO) localization. Proportion of VTs for which the distance between VT-SoO and vulnerable sites was <10 mm **(A)** (accurate localization of VT-SoO) and >20 mm **(B)** (inaccurate localization of VT-SoO). *P <.05 with respect to RVI_MIN_. Abbreviations as in Figure 6.

The identification of VT-SoO was considered accurate if the distance to VT-SoO was <10 mm and inaccurate if >20 mm. RVI showed the highest accuracy, with 13 of 18 VT-SoO located within 10 mm to the lowest RVI sites (72.2%) (Figure 7A) as well as the lowest inaccuracy rate, with only 1 VT-SoO located >20 mm from the lowest RVI sites (5.6%) (Figure 7B). Inaccurate identification of VT-SoO was significantly less likely to occur for lowest RVI than for longest AT and longest ARI (odds ratio 0.12; *P* = .035, χ^2^ for both markers) (Figure 7B).

### Interaction between RVI and other markers

RVI showed a positive correlation with ARI [cc 0.71 (0.59–0.76)] and RT [cc 0.52 (0.23–0.72)], and a weaker inverse correlation with AT [cc –0.26 (–0.45 to –0.07)], G_AT_ [cc –0.25 (–0.47 to –0.12)], and G_ARI_ [cc –0.26 (0.44–0.09)] (Supplementary Figure 2A).

Sites with lowest RVI partially overlapped with those showing shortest RT and ARI, with overlap of 35% (23%–42%) and 24% (12%–44%), respectively (Supplementary Figure 2B). Only 5% (0%–16%) of sites with lowest RVI were also identified as vulnerable because of longest AT. This increased to 13% (0%–21%) when considering as overlapping sites situated at a distance ≤2 mm. About 15% of sites showing lowest RVI also showed largest gradients of AT or ARI. This suggests that RVI captures electrophysiological vulnerability independently from standard activation-repolarization markers.

## Discussion

This is the first study to use state-of-the-art mapping technology to comprehensively evaluate RVI and alternative activation-repolarization markers in delineating the sites critical for VT establishment. The main results are as follows. (1) RVI identifies vulnerable regions that were within 10 mm of VT-SoO in 72.2% of VTs and >20 mm away in only 5.6% of VTs, with the closest vulnerable site located 5.1 (3.2–10.1) mm from the VT-SoO. (2) Inaccurate VT-SoO identification was significantly less frequent for lowest RVI than for longest AT and longest ARI. (3) Lowest RVI identifies vulnerable regions independent of other activation and repolarization markers, and it incorporates information from both local AT and ARI gradients, which identified critical regions at a (nonsignificantly) larger distance than lowest RVI.

RVI is based on a conceptual model of the critical relationship between activation and repolarization restitution properties, which was formerly defined by Coronel et al^6^ in an elegant animal study. Their study demonstrated that the critical parameter to differentiate block from the initiation of reentrant arrhythmia was the interval between the proximal RT of the premature beat and the arrival time of the premature wave at the distal side of the line of block. This represents the foundation of the RVI algorithm, first implemented by Child et al^7^ in a mechanistic proof-of-principle study. Results from retrospective analysis of noncontact mapping data in selective RV disorders^10^ and computational studies^8^^,^^9^ have provided support for the validity of the RVI concept. This study has assessed for the first time the RVI as a potential clinical tool to identify critical targets for ablation by utilizing state-of-the-art mapping technology in both RV and LV pathologies and comparing it to other activation-repolarization metrics of functional substrate. The results suggest that RVI could represent a useful metric to inform novel substrate ablation strategies.

Whereas other studies have focused on improving the delineation of the arrhythmogenic substrate with late potentials,^18^^,^^19^ metrics related to slow conduction, visualization of potential diastolic pathway, and characterization of channels using imaging,^2^^,^^20^ this study demonstrates that repolarization is critical for the identification of VT-SoO.

RVI performed similarly to, but independently of, local gradients of activation, an established marker of arrhythmia susceptibility,^21^ which is embedded in the RVI concept. A moderate correlation between RVI and local activation and repolarization gradients confirms the theoretical observation that RVI integrates information from both activation and repolarization dynamics.

### Current performance and future developments

Despite its solid theoretical underpinning, RVI was not significantly superior to other markers in the identification of VT-SoO. This may be partially due to lack of statistical power (n = 18) and both procedural and technological limitations. A critical aspect of RVI is the pacing protocol, with both cycle length and pacing site potentially affecting RVI maps.^8^ The importance of stimulating the tissue at a coupling interval short enough to engage conduction velocity restitution to unmask electrophysiological vulnerability is well recognized.^19^ It is possible that a more aggressive S_1_S_2_ pacing protocol could have provided more precise localization but at the risk of hemodynamic compromise and VT induction in these vulnerable patients. The pacing site affects both voltage^22^ and activation-repolarization properties,^23^ and pacing from multiple sites may improve RVI delineation of the arrhythmogenic substrate.^8^ This was not feasible due to time constraints during the procedure.

Importantly, in structurally abnormal hearts, multiple pathways may support different VTs, some of which may not be revealed during the procedure. This limits the extent by which any metric theoretically related to sites susceptible to reentry can be validated using information from the VTs mapped during the procedure. Validation by prospective studies using ablation to target all low RVI sites will be required in randomized controlled trials to test this physiological mapping approach vs current VT ablation strategies to determine VT recurrence, hospitalizations, and mortality.

### Study limitations

Although bespoke software solutions were implemented to analyze only beats with the same activation-repolarization sequence and semiautomatic correction was kept to a minimum to ensure reproducibility, repolarization variability during sequential mapping and the challenge of measuring activation/repolarization markers in diseased myocardium may have affected the results. Ultrafast noncontact mapping providing AT and RT within 1 single beat may represent a possible solution.^24^

Identification of VT-SoO with pacing maneuvers presents limitations inherent to electroanatomic mapping. Pace-mapping is a standard approach to identify the exit site of unstable VTs,^11^ but its accuracy can be affected by area of capture and functional block only present in VT. A previous study reported 82% sensitivity and 87% specificity in identifying the exit region by pace-mapping, with a 82% morphology match.^11^ Our cutoff value of 90% morphology match should provide slightly higher specificity. Although none of the mapped VTs were inducible after ablation, acute VT induction could not be consistently tested as procedural endpoint because of hemodynamic compromise in 6 patients. The recurrence rate was 50% after median follow-up of 16 months, which is in line with other studies.^1^ This is likely due to limitations of current ablation strategies in complex patients with hemodynamic compromise and presenting with multiple potential vulnerable sites for VT development, most of which may be concealed at the time of the procedure and may not be localized with entrainment, pace-mapping, or standard substrate mapping. This study did not set out to prospectively ablate low RVI sites, which will be the subject of future studies to assess whether this influences outcomes.

## Conclusion

The study data show that RVI identifies vulnerable regions that closely correlate with VT-SoO and suggest that activation-repolarization metrics may improve the delineation of the arrhythmogenic substrate and enable optimal substrate-based ablation without the risks of compromising patients with multiple VT inductions.
