# Supplementary material for: Evaluation of the reentry vulnerability index to predict ventricular tachycardia circuits using high-density contact mapping
Source: Heart Rhythm. 2020 Apr;17(4):576–83. doi: 10.1016/j.hrthm.2019.11.013 (PMC7105818; doi:10.1016/j.hrthm.2019.11.013)
Supplement: Supplemental Material [file mmc1.pdf]

SUPPLEMENTAL MATERIAL

Contents

Detailed Supplemental Methods 2

Detailed Data Analysis 2

Detailed Statistical Analysis 3

References 3

Supplementary Figures 7

Supplementary Figure 1 7

Supplementary Figure 2 8

Detailed Supplemental Methods

Detailed Data Analysis

Unipolar electrograms were recorded with band-pass filters set at 0.05-500 Hz and were exported along with anatomical data for bespoke off-line analysis in Matlab (Mathwork), which included stringent criteria for selecting only beats showing very similar activation/repolarization patterns. Although mapping systems should automatically ensure that only beats showing very similar surface ECG are recorded, the selection of beats to be included into the analysis was further refined. Unipolar electrograms were re-aligned on the pacing spike and the signal-averaged template of the surface ECG (12 leads in CARTO, 3 leads in Ensite Precision) of the recorded beat was generated. The unipolar electrograms recorded during a given beat were included only if the mean correlation coefficient between the surface ECG of that beat and the template was >0.85 for the entire signal, >0.90 within the QRS complex and >0.80 within the T-wave. This ensured that only beats with very similar activation and repolarization sequences were included.

As shown in Figure 2, AT was defined at the time of the minimum of the first derivative, dV/dt_{min}, and RT was defined at the maximum of the first derivative of the T-wave of the unipolar electrogram, dV/dt_{max}. This is the standard definition firstly proposed by Wyatt [1], which has become widely accepted and has been largely validated in animal [2]–[4], human [5] and theoretical [6]–[8] studies.

Activation recovery interval, ARI=RT-AT, was used as a surrogate for action potential duration. Annotation of unipolar electrograms was performed automatically within windows of interest. The window of interest for AT measurement started before and ended after the QRS complex of the surface ECG. The window of interest for RT encompassed the entire T-wave of the surface ECG, going from the earliest T-wave onset to the latest T-wave end across all leads.

Markers were revised using bespoke graphical user interfaces and corrected semi-automatically if needed. Semi-automatic correction consisted in performing automatic

annotation within manually adjusted windows of interest and it was limited to rare isolated outliers to ensure reproducibility. Furthermore, arbitrary annotation of AR and RT was prevented by the software.

Although measurements of AT and RT can be challenging, our bespoke algorithms have been optimized using expertise developed during more than 10 years of work in the field [8], [9], [18]–[22], [10]–[17].

Regarding validation of local repolarization time measurements, apart from the detailed and elegant studies mentioned before, we have recently demonstrated that repolarization time measured using our own algorithms provide an accurate estimation of the ventricular effective refractory period in 11 patients undergoing electrophysiological studies [23].

Detailed Statistical Analysis

Data are reported as median (1st quartile, 3rd quartile). The Wilcoxon signed-rank test, a non-parametric test for matched samples, was used for comparing distances between VT_{SO} and vulnerable sites identified by different markers. $P<0.05$ was considered as the threshold for significance. The spatial correlation between activation-repolarization markers, e.g. between RVI and AT, was quantified using the Spearman's correlation coefficient. The overlap between vulnerable sites identified by different markers (e.g. RVI_{MIN} and AT_{MAX}) was measured as the number of sites simultaneously identified as vulnerable by both markers (e.g. sites for which $RVI<5^{th}$ percentile of RVI values and $AT>95^{th}$ of AT values), divided by the average number of vulnerable sites identified by the two markers.

References

[1] R. F. Wyatt, M. J. Burgess, A. K. Evans, R. L. Lux, J. A. Abildskov, and T. Tsutsumi, "Estimation of ventricular transmembrane action potential durations and repolarization times from unipolar electrograms," *Am. J. Cardiol.*, vol. 47, p. 488, Feb. 1981.

[2] R. Coronel *et al.*, “Monophasic action potentials and activation recovery intervals as measures of ventricular action potential duration: Experimental evidence to resolve some controversies,” *Heart Rhythm*, vol. 3, no. 9, pp. 1043–1050, 2006.

[3] C. K. Millar, F. A. Kralios, and R. L. Lux, “Correlation between refractory periods and activation-recovery intervals from electrograms: Effects of rate and adrenergic interventions,” *Circulation*, vol. 72, no. 6, pp. 1372–1379, 1985.

[4] C. W. Haws and R. L. Lux, “Correlation between in vivo transmembrane action potential durations and activation-recovery intervals from electrograms. Effects of interventions that alter repolarization time.,” *Circulation*, vol. 81, no. 1, pp. 281–288, 1990.

[5] M. Chinushi *et al.*, “Correlation Between the Effective Refractory Period and Activation-Recovery Interval Calculated From the Intracardiac Unipolar Electrogram of Humans With and Without dl-Sotalol Treatment,” *Jpn. Circ. J.*, vol. 65, no. 8, pp. 702–706, 2001.

[6] B. M. Steinhaus, “Estimating cardiac transmembrane activation and recovery times from unipolar and bipolar extracellular electrograms: a simulation study.,” *Circ. Res.*, vol. 64, no. 3, pp. 449–462, 1989.

[7] M. Potse, A. Vinet, T. Opthof, and R. Coronel, “Validation of a simple model for the morphology of the T wave in unipolar electrograms.,” *Am. J. Physiol. Heart Circ. Physiol.*, vol. 297, no. 2, pp. H792–H801, 2009.

[8] M. Orini, P. Taggart, and P. D. Lambiase, “In vivo human sock-mapping validation of a simple model that explains unipolar electrogram morphology in relation to conduction-repolarization dynamics,” *J. Cardiovasc. Electrophysiol.*, vol. 29, no. 7, pp. 990–997, Jul. 2018.

[9] M. Orini, N. Srinivasan, P. Taggart, and P. Lambiase, “Reliability of APD-restitution slope measurements: Quantification and methodological comparison,” in *Computing in Cardiology*, 2015, vol. 42, pp. 545–548.

[10] M. Orini *et al.*, “Noninvasive Mapping of the Electrophysiological Substrate in Cardiac Amyloidosis and Its Relationship to Structural Abnormalities,” *J. Am. Heart Assoc.*, vol. 8, no. 18, Sep. 2019.

[11] N. T. Srinivasan *et al.*, “Differences in the upslope of the precordial body surface ECG T wave reflect right to left dispersion of repolarization in the intact human heart,” *Heart Rhythm*, vol. 16, no. 6, pp. 943–951, Dec. 2019.

[12] M. Orini *et al.*, “Mechanistic insights from targeted molecular profiling of repolarization alternans in the intact human heart,” *Europace*, vol. 21, no. 6, pp. 981–989, Feb. 2019.

[13] A. J. Graham *et al.*, “Simultaneous Comparison of Electrocardiographic Imaging and Epicardial Contact Mapping in Structural Heart Disease,” *Circ. Arrhythm. Electrophysiol.*, vol. 12, no. 4, p. e007120, Apr. 2019.

[14] M. Orini, P. Taggart, N. Srinivasan, M. Hayward, and P. D. Lambiase, “Interactions between activation and repolarization restitution properties in the intact human heart: In-vivo whole-heart data and mathematical description,” *PLoS One*, vol. 11, no. 9, 2016.

[15] M. Orini *et al.*, “Comparative evaluation of methodologies for T-wave alternans mapping in electrograms,” *IEEE Trans. Biomed. Eng.*, vol. 61, no. 2, pp. 308–316, Feb. 2014.

[16] M. Orini, L. Citi, B. M. B. M. Hanson, P. Taggart, and P. D. P. D. P. D. Lambiase, “Characterization of the causal interactions between depolarization and repolarization temporal changes in unipolar electrograms,” in *Computing in Cardiology*, 2013, vol. 40, no. v, pp. 719–722.

[17] M. Orini, B. Hanson, P. Taggart, and P. Lambiase, “Detection of transient, regional cardiac repolarization alternans by time-frequency analysis of synthetic electrograms,” in *Proceedings of the Annual International Conference of the IEEE Engineering in Medicine and Biology Society, EMBS*, 2013, vol. 2013, pp. 3773–3776.

[18] S. Van Duijvenboden, M. Orini, P. Taggart, and B. Hanson, “Accuracy of measurements

derived from intracardiac unipolar electrograms: A simulation study," in *Proceedings of the Annual International Conference of the IEEE Engineering in Medicine and Biology Society, EMBS*, 2015, vol. 2015-Novem, pp. 76–79.

[19] M. Orini, P. Taggart, M. Hayward, and P. Lambiase, "On how 2/1 conduction block can induce T-wave alternans in the unipolar intracavitary electrogram: Modelling in-vivo human recordings from an ischemic heart," in *Proceedings of the Annual International Conference of the IEEE Engineering in Medicine and Biology Society, EMBS*, 2015, vol. 2015-Novem, pp. 5676–5679.

[20] M. Orini, S. Van Duijvenboden, N. Srinivasan, M. Finlay, P. Taggart, and P. D. Lambiase, "Theoretical assessment of a repolarization time marker based on the intracardiac bipolar electrogram," in *Computing in Cardiology*, 2017, vol. 44, pp. 1–4.

[21] X. Zhou *et al.*, "In Vivo and in Silico Investigation into Mechanisms of Frequency Dependence of Repolarization Alternans in Human Ventricular Cardiomyocytes," *Circ. Res.*, vol. 118, no. 2, pp. 266–278, 2016.

[22] C. A. Martin *et al.*, "Assessment of a conduction-repolarisation metric to predict Arrhythmogenesis in right ventricular disorders," *Int. J. Cardiol.*, vol. 271, pp. 75–80, May 2018.

[23] M. Orini, N. T. Srinivasan, A. Graham, P. Taggart, and P. Lambiase, "Further evidence on how to measure local repolarization time using intracardiac unipolar electrograms in the intact human heart," *Circ. Arrhythm. Electrophysiol.*, no. In Press, 2019.

Supplementary Figures

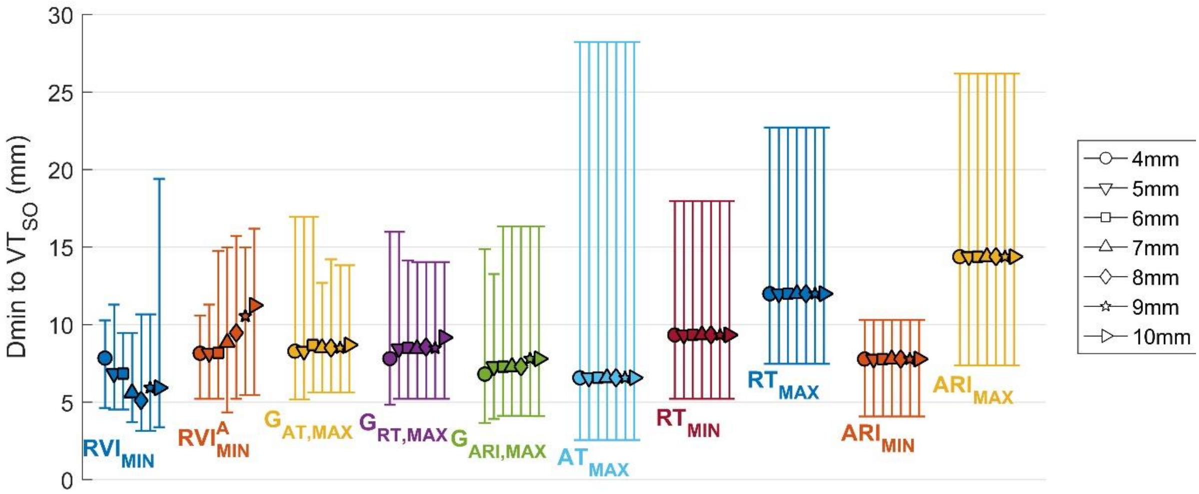

Supplementary Figure 1**:** Distance between the VT site of origin (VT-SoO) and the nearest vulnerable sites identified by low RVI (RVI_{MIN}), low RVI-modified (RVI_{MIN}^{A}), large gradient of activation time ($G_{AT,MAX}$), large gradient of repolarization time ($G_{RT,MAX}$), large gradient of activation recovery interval ($G_{ARI,MAX}$), long activation time (AT_{MAX}), short repolarization time (RT_{MIN}), long RT (RT_{MAX}), short activation recovery interval (ARI_{MIN}) and long ARI (ARI_{MAX}). Markers indicate the median of minimum distances and bars span the 1st-3rd quartile interval (across VTs). A different symbol is used to show results obtained using different search radius to define neighbouring sites. At each cardiac site, RVI-modified was measured as the mean RT_P-AT_D interval instead of the minimum RT_P-AT_D interval.

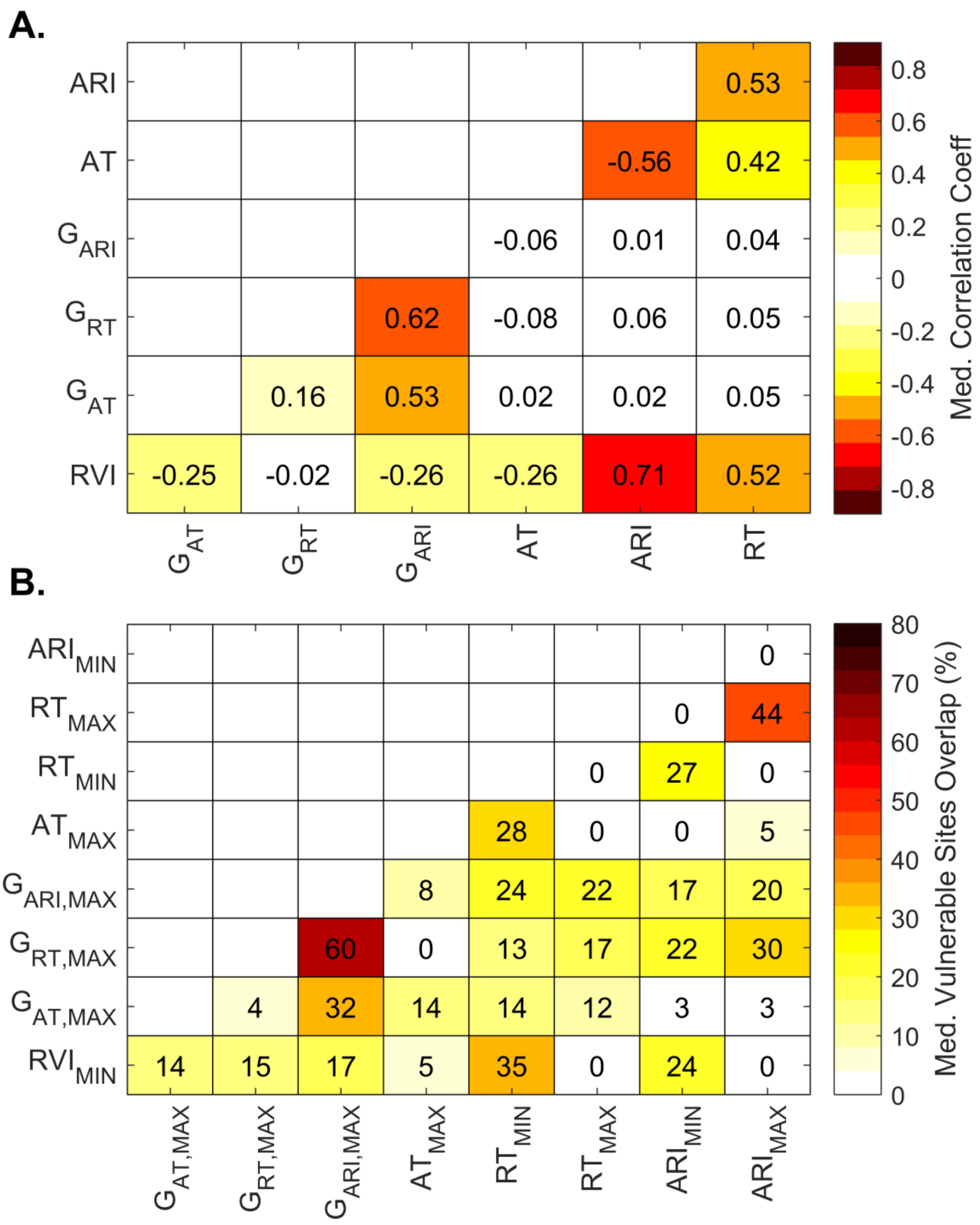

Supplementary Figure 2**: Interactions between activation-repolarization metrics.** A: Matrix showing the median correlation coefficient between all the markers included in the study: re-entry vulnerability index (RVI), activation time (AT), repolarization time (RT), activation recovery interval (ARI), local gradients of AT (G_{AT}), local gradients of ARI (G_{ARI}), local gradients of RT (G_{RT}). B: Matrix showing the median overlap between vulnerable regions identified by any pair of markers included in the study. The sub-index “MIN” and “MAX” indicate

that the vulnerable sites have been identified as belonging to the (0th-5th) or (95th-100th) percentile interval, respectively. As an example, cell (1,3) shows that the median number of sites simultaneously identified as vulnerable to re-entry by low RVI and large G_{ARI} was 17% of the total number of sites identified as vulnerable by the two markers.
